# Supplementary material for: Hybrid Models and Biological Model Reduction with PyDSTool
Source: PLoS Comput Biol. 2012 Aug 9;8(8):e1002628. doi: 10.1371/journal.pcbi.1002628 (PMC3415397; doi:10.1371/journal.pcbi.1002628)
Supplement: Text S4 — Complete source code for the PyDSTool package (version 0.88.120504). Includes API documentation and help files linking to web pages. This file is identical to the current public release on Sourceforge.net. (ZIP) [file pcbi.1002628.s004.zip › PyDSTool/html/PyDSTool.Toolbox.optimizers.step.partial_step-pysrc.html]

xml version="1.0" encoding="ascii"?


PyDSTool.Toolbox.optimizers.step.partial\_step


| Home | Trees | Indices | Help | PyDSTool |
| --- | --- | --- | --- | --- |

Package PyDSTool ::
Package Toolbox ::
Package optimizers ::
Package step ::
Module partial\_step

[hide private]

[frames] | no frames]

# Source Code for Module PyDSTool.Toolbox.optimizers.step.partial\_step

```
 1   
 2  # Matthieu Brucher 
 3  # Last Change : 2007-08-10 23:15 
 4   
 5  """ 
 6  Computes a partial step for a specific function at a specific point, acting like a decorator for other steps 
 7  """ 
 8   
 9  import random 
10  import numpy 
11   


12 -class PartialStep(object):


13    """ 
14    A partial step 
15    """ 


16 -  def __init__(self, step, nb_chunks, indice = None):


17      """ 
18      Allows only a part of the set of parameters to be updated 
19        - step is the step that will be decorated 
20        - nb_chunks is the number of chunks in the parameter set 
21        - indice is the chunk to update each time. if None, a random indice is drawn each time 
22      """ 
23      self.step = step 
24      self.nb_chunks = nb_chunks 
25      self.indice = indice

26   


27 -  def __call__(self, function, point, state):


28      """ 
29      Computes a step based on a function and a point 
30      """ 
31      step = self.step(function, point, state) 
32      state['old_direction'] = step 
33      if self.indice is None: 
34        indice = random.randint(0, self.nb_chunks - 1) 
35      else: 
36       indice = self.indice 
37      new_step = numpy.zeros(step.shape) 
38      new_step.shape = (self.nb_chunks, -1) 
39      step.shape = (self.nb_chunks, -1) 
40      new_step[indice] = step[indice] 
41   
42      new_step.shape = -1 
43      step.shape = -1 
44      state['direction'] = new_step 
45      return new_step

46
```

  


| Home | Trees | Indices | Help | PyDSTool |
| --- | --- | --- | --- | --- |

|  |  |
| --- | --- |
| Generated by Epydoc 3.0.1 on Fri May 4 15:24:16 2012 | http://epydoc.sourceforge.net |
